# High production of carotenoids by the green microalga *Asterarcys quadricellulare* PUMCC 5.1.1 under optimized culture conditions

**DOI:** 10.1371/journal.pone.0221930

**Published:** 2019-09-06

**Authors:** Davinder Pal Singh, Jasvirinder Singh Khattar, Alka Rajput, Rajni Chaudhary, Ramsarup Singh

**Affiliations:** 1 Department of Botany, Punjabi University, Patiala, Punjab, India; 2 Department of Biotechnology, Punjabi University, Patiala, Punjab, India; Universite Paris-Sud, FRANCE

## Abstract

Since carotenoids are important as natural colorants, antioxidants, neutraceutics and pharmaceutics, the aim of the present study was to find a new good source of these pigments. We hereby report a green microalga *Asterarcys quadricellulare* PUMCC 5.1.1 as a new and good producer of carotenoids. The organism produced 35±1.75 μg carotenoids mg^−1^ dry biomass during stationary phase in control cultures. The growth and carotenoids production by the test microalga were optimized by varying nutrient growth media, pH, nitrogen and phosphate source, salinity, light quality, intensity and duration. The optimized conditions for carotenoid production were: Bold basal (BB) medium with pH 8.5, containing with10 mM nitrate, 3.5 mM phosphate and 0.17 mM salinity and illuminated with blue light with 60 μmol m^-2^ s^-1^ photon flux light intensity. Cultivation of cultures in the above mentioned optimized conditions resulted in nearly 3.0 fold increase in carotenoid production compared to the control cultures grown in unmodified BB medium. Using HPTLC, four carotenoids have been identified as β-carotene, lutein, astaxanthin and canthaxanthin. Further, carotenoids were also separated and purified by flash chromatography and the amounts of purified carotenoids were determined by HPLC. The organism produced 47.0, 28.7, 15.5 and 14.0 μg β-carotene, lutein, astaxanthin and canthaxanthin mg^−1^ dry biomass, respectively, under optimized conditions. The amount of total carotenoids (118 μg mg^-1^ dry biomass) produced by *Asterarcys quadricellulare* PUMCC 5.1.1 under optimized culture conditions was significantly higher than control cultures. Thus, this microalgal strain is a promising candidate for carotenoid production at commercial level.

## Introduction

Colour plays a special role in selection of food before consumption. Colorants are deliberately added to the food items to enhance their visual appeal [1, 2]. Recently, increasing evidence on harmful effects of synthetic colours has compelled regulatory agencies across the world to drastically prune the list of permitted synthetic food colours [3, 4]. Since synthetic colours are causing several health problems, thus there is a worldwide attention for looking safe colourants from natural sources. Carotenoids are a group of natural, fat soluble pigments responsible for the yellow, orange and red colour especially in fruits, vegetables and flowers [5]. These are also distributed in certain roots, seaweeds, invertebrates, fishes, crustacean shells, skin, birds, bacteria, fungi and microalgae [6–8].

Carotenoids are of great interest in scientific studies because of their wide distribution, diverse functions and interesting properties [9, 10]. Epidemiological and experimental evidence indicate that dietary carotenoids inhibit the onset of diseases such as arteriosclerosis, cataract, age related macular degeneration, multiple sclerosis, cancer and arthritis. These also have anti-aging, anticancer and immune modulatory properties [8, 10, 11]. It has been reported that carotenoids extracted from *Chlorella ellipsoidea* and *Chlorella vulgaris* inhibited colon cancer development [12]. Furthermore, astaxanthin obtained from *Haematococcus pluvialis* decreased expression of cyclin D1 but increased that of p53 and some cyclin kinase inhibitors of colon cancer cell lines [8, 13]. Recent studies have demonstrated the efficacy of carotenoids against HIV infection [14]. Rising consumer awareness about the health benefits of many carotenoids, ongoing trend favoring healthy and natural food products have benefited carotenoids industry. The global carotenoid market was estimated to be ~1.5 billion USD in 2017, and is projected to increase to ~2 billion USD by 2024, at a compound annual growth rate of 5.72% from 2016 to 2024. β-carotene holds the largest share of market followed by astaxanthin (http://www.bccresearch.com; accessed on 20.01.2019).

Microalgae are one of the major sources of natural carotenoids. Their fast and active growth, short life cycle, active isoprenoid metabolism precursors for the carotenogenic pathway, and adequate storage capacity make these as ideal cell factories for the production of high value carotenoids [11]. Further bioimaging technology assists easily to estimate the distribution of carotenoids in the cells of microalgae [15]. Microalgae have ability to synthesize high amounts of mixture of carotenoids such as astaxanthin, β-carotene, canthaxanthin and echinenone under unfavorable conditions [16]. Among the microalgae, *Botryococcus braunii*, *Chlorella* sps., *Chlorococcum* sp., *Coelastrella striolata*, *Haematococcus pluvialis*, *Dunaliella salina*, *Nanochloropsis* sps., *Scenedesmus* sps, *Spirulina platensis*, and some diatoms are known for production of β-carotene, lutein, canthaxanthin, astaxanthin and fucoxanthin [1, 17–19]. The diverse microalgal genetic pool still remains to be fully evaluated for carotenoid production. The present market of carotenoids is mainly based on astaxanthin and β-carotene but other carotenoids such as lutein and canthaxanthin still have not much been exploited. Among the microalgae, *Dunaliella salina* and *Haematococcus pluvialis* are widely studied taxa for β-carotene and astaxanthin production on commercial scale, respectively [19, 20]. Though these microalgae are highly productive but culturing and maintenance of these strains under native environmental conditions is very difficult. To overcome this problem, regional microalgal flora should be explored for potential adaptation to industrial scale production. Reports are available suggesting that culture conditions like pH of the medium, light intensity and quality, concentration of the nitrogen source, sodium chloride and phosphorus affect carotenoid production in algae [21–23]. These reports indicate that carotenoid production by microalgae can be enhanced by manipulating culture conditions. Keeping this in mind, the present investigation was aimed to look for new high carotenoid producing microalgal strains and to optimize culture conditions for further enhancement of the pigment.

## Material and methods

### Organism and culture conditions

The microalga *Asterarcys quadricellulare* PUMCC 5.1.1 was collected from fresh water of Kedarnath (30° 44' 24.97" N; 79° 4' 40.67"), Uttrakhand, India, identified and raised to axenic cultures through plating techniques by our laboratory [24]. Images of the organism were captured through a light microscope (LEICA DM 4000 B LED, Germany). The strain has been deposited in Microbial Culture Collection, Centre for Conservation and Utilization of Blue Green Algae (CCUBGA), ICAR-Indian Agriculture Research Institute (IARI), New Delhi with strain number MCC 42. The isolate was propagated in Bold Basal medium [25] which contained the following ingredients (L^-1^): NaNO_3_ (2.5 gm), MgSO_4_.7H_2_O (0.75 gm), NaCl (0.25 gm), K_2_HPO_4_ (0.75 gm), KH_2_PO_4_ (1.75 gm), CaCl_2_.2H_2_O (0.25 gm), ZnSO_4_.7H_2_O (8.82 mg), MnCl_2_.4H_2_O (1.44 mg), MoO_3_ (0.71 mg) CuSO_4_.5H_2_O (1.57 mg), CO(NO_3_)_2_.6H_2_O (0.49 mg), H_3_BO_3_ (1.14 mg), EDTA (5.0 mg), KOH (3.1 mg), FeSO_4_.7H_2_O (4.98 mg) and pH adjusted to 7.5. The cultures were incubated in the culture room at 28±2°C under white light with intensity 40 μmol m^-2^ s^-1^ photon flux (μE) at the surface of culture vessels with 14 h light and 10 h dark period.

### Molecular identification

Morphological based identification was further confirmed by 18S rRNA gene sequence analysis. Total genomic DNA was extracted following Moncalvo et al. [26]. The cell pellet obtained after centrifugation at 10,000xg for 10 min was suspended in 500 μL of DNA extraction buffer (200 mM Tris HCL, pH 8.5, 250 mM NaCl, 25 mM EDTA and 0.5% SDS) at 60°C, incubated in a water bath for 90 min and genomic DNA was extracted with 350 μL of ice cold phenol: chloroform: isoamyl alcohol (25:24:1). Extracted DNA was precipitated with 250 μL cold isopropanol and centrifuged. The obtained DNA pellet washed with 70% ethanol, air dried, resuspended in 50 μL TE buffer (10 mM Tris HCL, pH 8.0 and 1 mM EDTA) and stored at -20°C. The 18S rRNA gene (fragment of 960 bp) of the isolate was amplified by using primers 18SU 467F ATCCAAGGAAGGCAGCAGGC and 18SU 1310R CTCCACCAACTAAGAACGGC [27]. Total PCR reaction mixture was consisted of 2X buffer mixture, 10 μL each of forward and reverse primer and 50 ng template DNA. Gene amplification was done by initial denaturation at 94°C for 4 min, followed by 35 cycles of 1 min at 94°C, 1 min at 52°C and 2 min at 72°C and final extension at 72°C for 8 min. The gel purified PCR product was obtained using QIA quick PCR purification kit (Qiagen GmBh, Germany). The gene sequence was done by using AB1310 automatic DNA sequencer (Applied Biosystem, USA). The obtained 960 bp gene sequence was analyzed using the gapped BLASTn (http://www.ncbi.nim.nih.gov/blast) search algorithm and aligned to the near neighbours. The phylogenetic tree was constructed using MEGA 6.00 software package [28].

### Screening of microalgae for carotenoid production

Forty randomly selected strains of microalgae isolated from diverse habitats were screened for their growth and carotenoid production. Experiment was set up in 250 mL Erlenmeyer flasks containing 100 mL BG-11 medium. Exponentially growing 6–8 day old cultures of each strain were washed twice with sterilized double distilled water and added in flasks to get initial absorbance 0.1 at 720 nm using UV-visble specterophotmeter (Model 1280; Shimadzu, Japan). At regular interval of 2 day, 20 mL cultures were harvested and growth (A_720_) and amount of total carotenoids were determined. The growth rate was calculated using formula:

$$\mu =\frac{3.3\;(\log_{10}N‐\log_{10}N_{0})}{t}$$

where μ: growth rate, N and N_0_: final and initial growth, respectively, t: time (h) interval between final and initial growth.

### Optimization of conditions for growth and carotenoid production

The growth of test organism was studied and compared in five nutrient media viz. BG-11 [29], Allen and Arnon [30], Bold Basal [25], Chu-10 [31] and Hoagland medium [32] to select the best nutrient medium for growth and carotenoid production. The experiment was conducted in 250 mL culture flasks containing 100 mL culture medium as explained above. On day 24, the culture was divided into two parts (Part I: 30 and Part II: 70 mL). Biomass from each part was harvested by centrifugation at 5,000×g for 10 min and obtained cell pellet was washed twice with double distilled water. Biomass pellet from Part I was oven dried at 70°C for 24 h and the weight of dry biomass was determined. Biomass pellet from Part II was used for the determination of carotenoids. The Bold basal medium supported maximum growth and carotenoid production and thus was selected for further study. The growth of and carotenoid production by the organism in this medium were further optimized by varying pH (5.5, 6.5, 7.5, 8.5, 9.5 and 10.5), temperature (18, 25, 30, 35 and 40°C), nitrogen (NaNO_3_; 3–40 mM) and phosphate (K_2_HPO_4_; 0.4–5.2 mM) source, salinity (NaCl; 0.04–0.85 mM) of selected medium and by changing quality, duration and intensity of light. The effect of continuous and discontinuous light was studied by incubating the cultures in continuous light without any dark period and 14 h light followed by 10 h dark, respectively. Five light intensities such as 10, 20, 40 60 and 80 μE were chosen to study effect of intensity of light on growth and carotenoid production. The effect of quality of light on growth and carotenoid production was studied by illuminating the culture vessels wrapped with the cellophane papers of red, blue, green and yellow colours [33]. The source of light was adjusted in such a way that each culture received same light intensity.

### Extraction and quantification of carotenoids

The amount of total carotenoids was estimated following the method of Lichtenthaler and Buschmann [34]. Two hundred mL of homogenous suspension of cultures was centrifuged at 5000xg for 10 min, and the cell pellet obtained was resuspended in 20 mL of acetone. The solvent biomass mixture was incubated at 50°C in a water bath for 2 h with manual shaking after every 10 min. The mixture was centrifuged at 5,000xg and absorbance of supernatant was noted at 661, 644 and 470 nm using UV-Visible spectrophotometer (Model 1280, Shimadzu, Japan). The amount of total carotenoids was determined using following equations:

$$Chlorophyll\;a\;(\mu g\;{mL}^{-1})=(11.24\;A_{660})–(2.04\;A_{645})$$

$${Chlorophyll\;b\;(\mu g\;mL}^{-1})=(20.13\;A_{645})–(4.19\;A_{660})$$

$${Carotenoids\;(\mu g\;mL}^{-1})=\frac{1000\;x\;(A_{470}-1.90\;Chl\;a-63.14\;Chl\;b)}{214}$$

Where A_660_, A_645_ and A_470_ represent absorbance at 660 nm, 645 nm and 470 nm, respectively. Simultaneously dry weight biomass of the cultures was noted and amount of carotenoids was equalized on dry weight basis.

### Identification and purification of carotenoids

The extract obtained above was processed on the automated HPTLC system (CAMAG Linomat 5 "Linomat 5–200109" S/N 200109 (1.00.13) for the separation and identification of carotenoids. Ready to use silica gel F_254_ TLC sheets (Merck, India) were activated by blowing hot air for 5–10 min and placed in the automatic sample applicator. The HPTLC was programmed to automatically load 10 μL of extract on one end of the TLC plate in band form using specialized Hamilton syringe. Mixture of petroleum ether: cyclohexane: ethyl acetate: acetone and methanol in ratio of 60:16:10:10:4 was used as a separating solvent. The plate was developed in the automated developing chamber (CAMAG) until the solvent front reached the maximum distance (7.5 cm distance in a typical 10 x 10 cm plate). The developed plate was dried with a plate drier and subjected to analysis using software CAMAG TLC Scanner "Scanner-200208" S/N 200208 (2.01.02). All tracks in the plate were scanned at user-defined wavelength and individual R_f_ values of peaks were obtained and identified by comparing R_f_ values of canthaxanthin, astaxanthin, lutein and β-carotene standards of carotenoids run parallel with unknown samples [35].

The carotenoids were also purified by Flash Column Chromatography (Biotage Isolera 1, Switzerland). Biotage (SNAP-10 gm) column filled with silica gel grade 60–120 mesh size was gradiently run with mobile phase composed of petroleum ether (A) and acetone (B) at flow rate of 8.0 mL min^-1^. The elution gradient of solvent was as follows: 0–10 min, 82% A and 18% B; 10–16 min, 79% A and 21% B; 16–22 min, 68% A and 32% B; 22–28 min 53% A and 47% B. The absorbance of the fractions was monitored at 254 nm and 280 nm by the UV detector. In the separation process, the eluent from the column was collected with the fraction collector set at 8 mL for each tube. Each fraction was dried under reduced pressure using vacuum evaporator. Each dried fraction was dissolved in acetone and analyzed by HPTLC as described above. Analysis of these fractions was compared with the known carotenoid standards such as canthaxanthin, astaxanthin, lutein and β-carotene. The identification of carotenoids in the fractions containing the above carotenoids was reconfirmed by HPLC (Waters 2707, Massachuetts, USA). The conditions of HPLC were: column; C_18_ column (length 250 mm, diameter 4.0 mm, particle size 5 μm, temperature 30 ^o^C), solvent; dichloromethane: acetonitrile: methanol (20:70:10, v/v/v), flow rate; 1.0 mL min^-1^; detector: PDA and volume of sample injected: 5 μL. The column was run in isocratic mode with the solvent for 25 min. The chromatograms were analyzed employing Empower 2 software and the amount of carotenoids was determined from the peak area of each identified carotenoid.

### Chemicals

All chemicals used during the present study were obtained from SD Fine Chemical Limited, Mumbai and Merck, India. Standards of carotenoids were purchased from Sigma-Aldrich Co., USA.

### Statistics

All the data are the average of three independent experiments ± standard deviation (SD). Data were statistically analyzed by one-way analysis of variance and Tukey’s post hoc significance difference test. All statistical analyses were tested against the probability value at 95% confidence level (p<0.05) using Graph Pad Prism 6.0 version (www.graphpad.com).

## Results

### Selection and identification of the microalga

**Fig 1:**
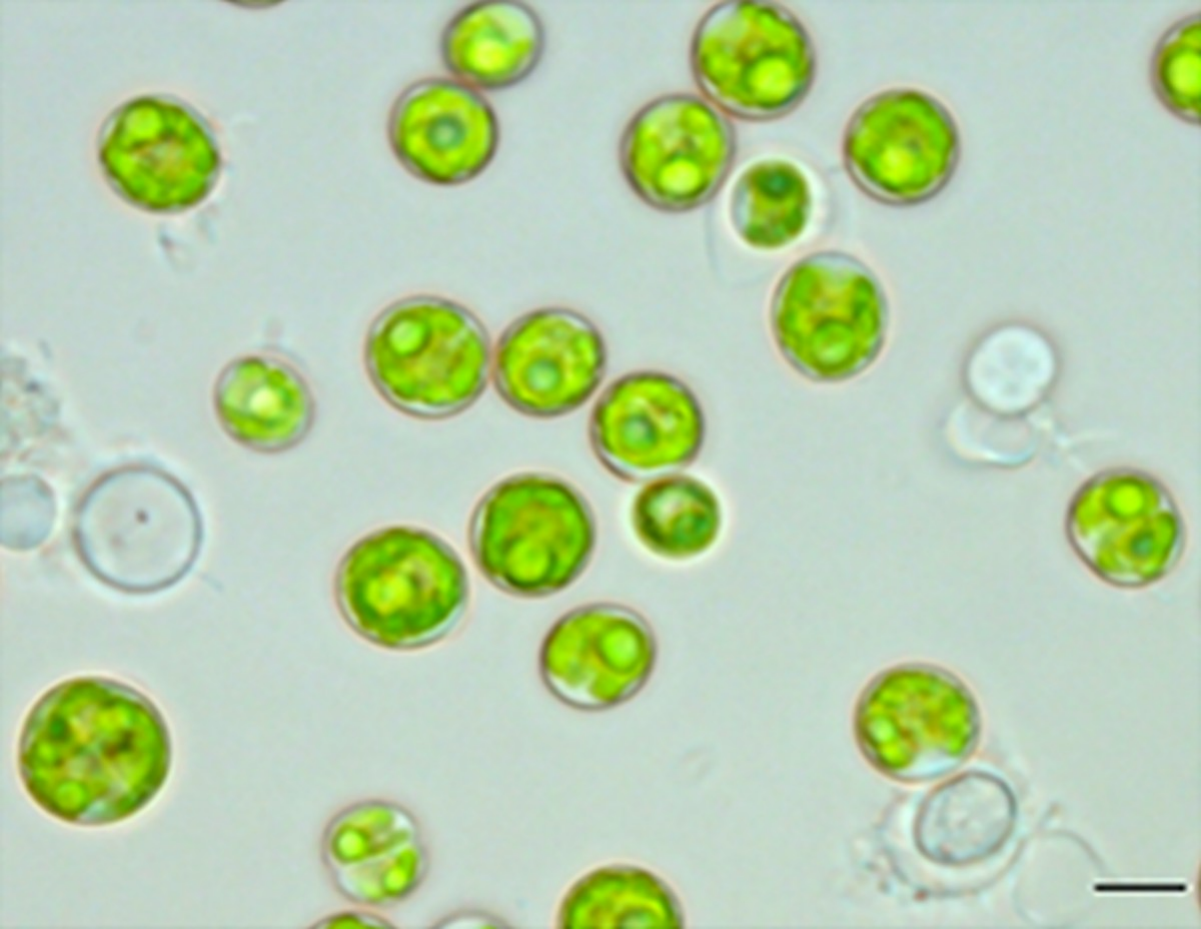
Photomicrograph of *Asterarcys quadricellulare* Scale bar: 10 μm. Based on morphological characters, this microalga was identified as *Asterarcys quadricellulare* and belongs to class Chlorophyceae, order Sphaeropleales, family Scenedesmaceae and sub-family Coelastoideae of group Chlorophyta.

In preliminary experiments, 40 microalgal strains representing *Chlorella*, *Chlorococcum*, *Scenedesmus*, *Desmodesmus*, *Coelastrum* and *Asterarcys* were screened for carotenoid production by growing them in BG 11 medium. Of these, *Asterarcys* strain FKN 40 exhibited maximum growth (μ = 0.0083 h^-1^) and produced maximum amount of carotenoids equivalent to 28.3±1.2 μg carotenoids mg^-1^ dry weight biomass on 8 d (S1 Table) and was selected for the present study. This isolate is non-motile with three-dimensionally arranged coenobium. The diameter of the coenobium varied from 3 μm to 20 μm depending on the growth stage. The coenobium consisted of generally 2 to 4 or more cells within a spherical mucilage envelope. Cells are ovoid in shape having single net-shaped chloroplast with pyrenoid in each cell (Fig 1).

**Fig 2:**
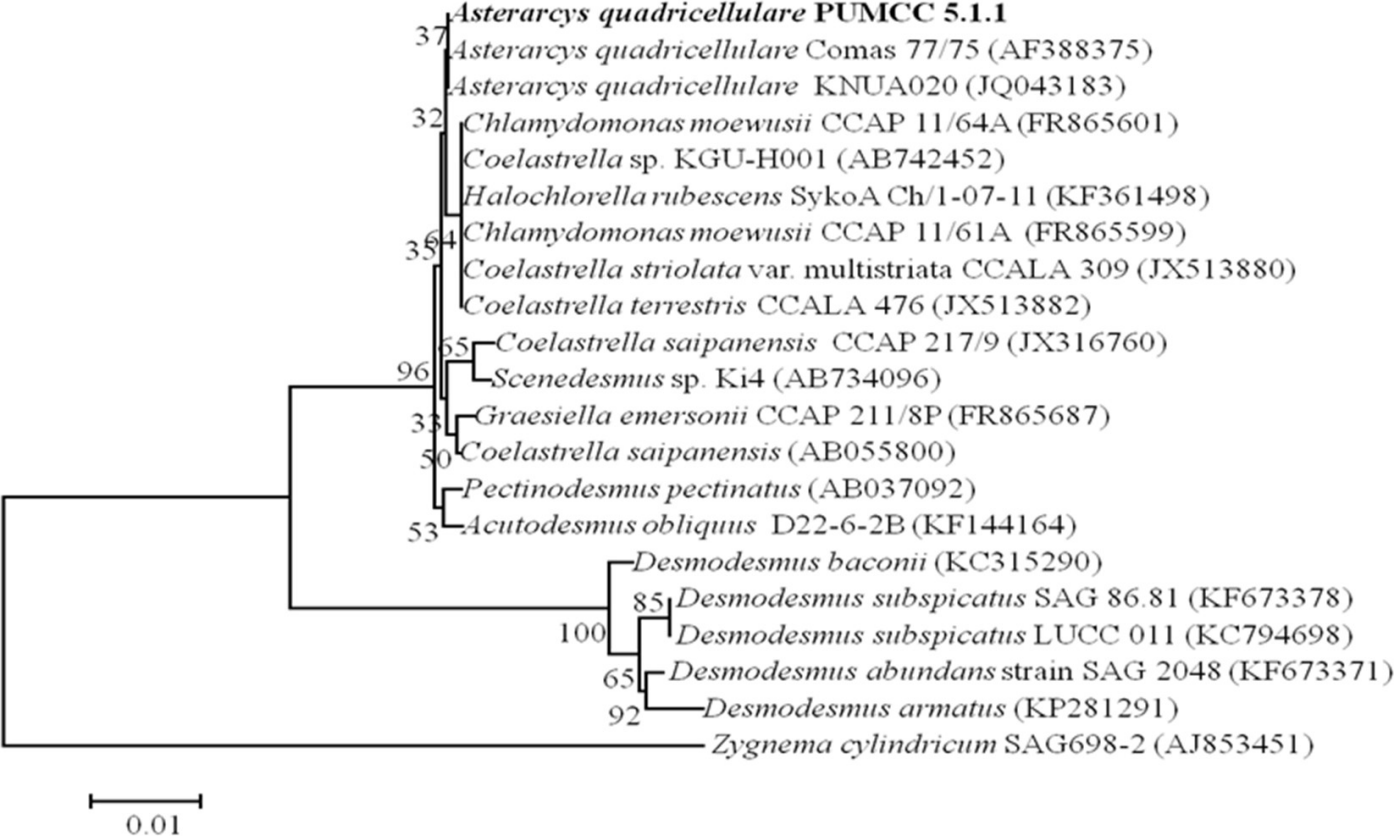
Phylogenetic tree showing relationship of *Asterarcys quadricellulare* with closely related taxa based on partial 18S rRNA gene sequence.

The identity of the test microalga was further confirmed by molecular characterization using partial 18S rRNA gene sequence analysis. The phylogenetic tree generated by aligning 18S rRNA gene sequence (960 bp) of test organism with sequences of microalgal strains obtained from NCBI GenBank showed that strain *Asterarcys quadricellulare* FKN 40 showed 100% similarity with *Asterarcys quadricellulare* Comas 75/76 studied by other worker (Fig 2). The nucleotide sequence obtained during the present study has been deposited in NCBI GenBank database with accession number KT151952. The isolate was named as *Asterarcys quadricellulare* PUMCC 5.1.1.

### Growth of and carotenoid production by *Asterarcys quadricellulare*

**Fig 3:**
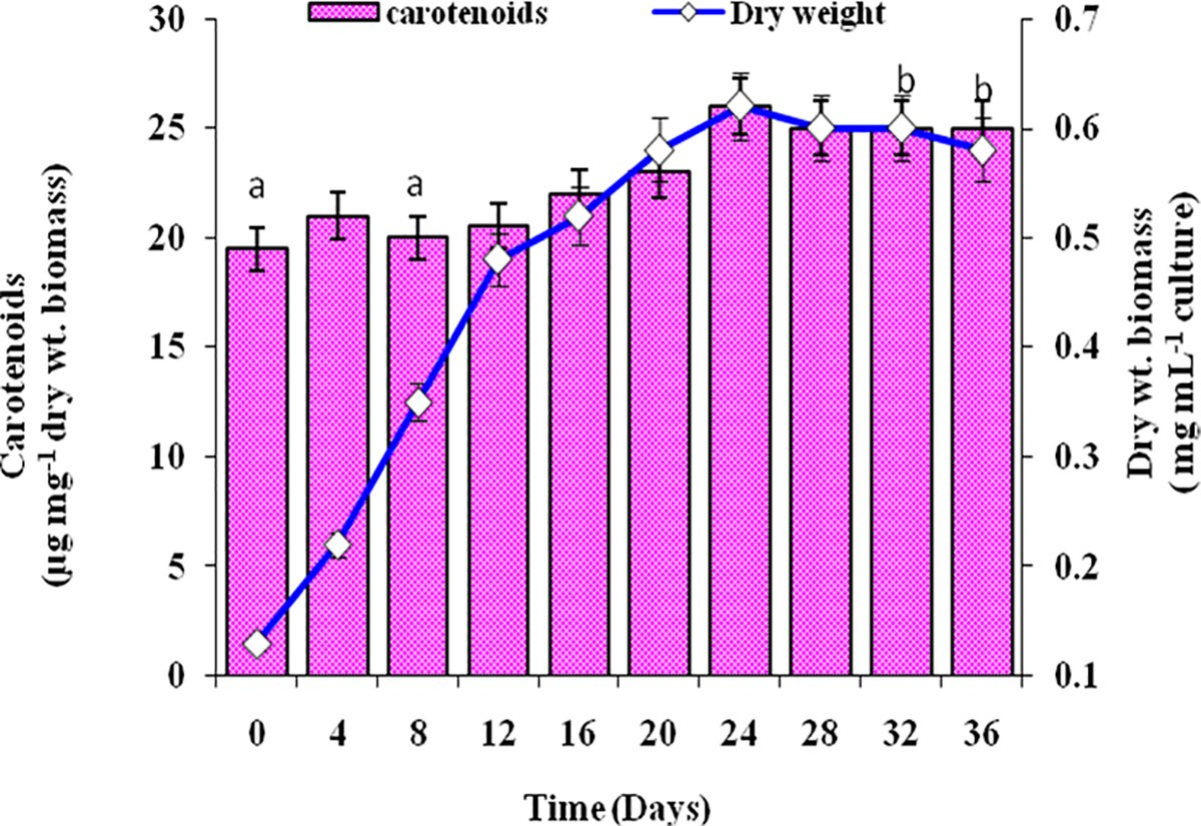
Growth (dry weight biomas) and carotenoid production by *Asterarcys quadricellulare* in BG-11 medium with time at 28 ^o^C. Light intensity 40 μE; 14:10 light:dark period. Data with same alphabet are not significantly different from each other at 95% confidence level (p ≤ 0.05). n: 6, error bar: SD.

**Fig 4:**
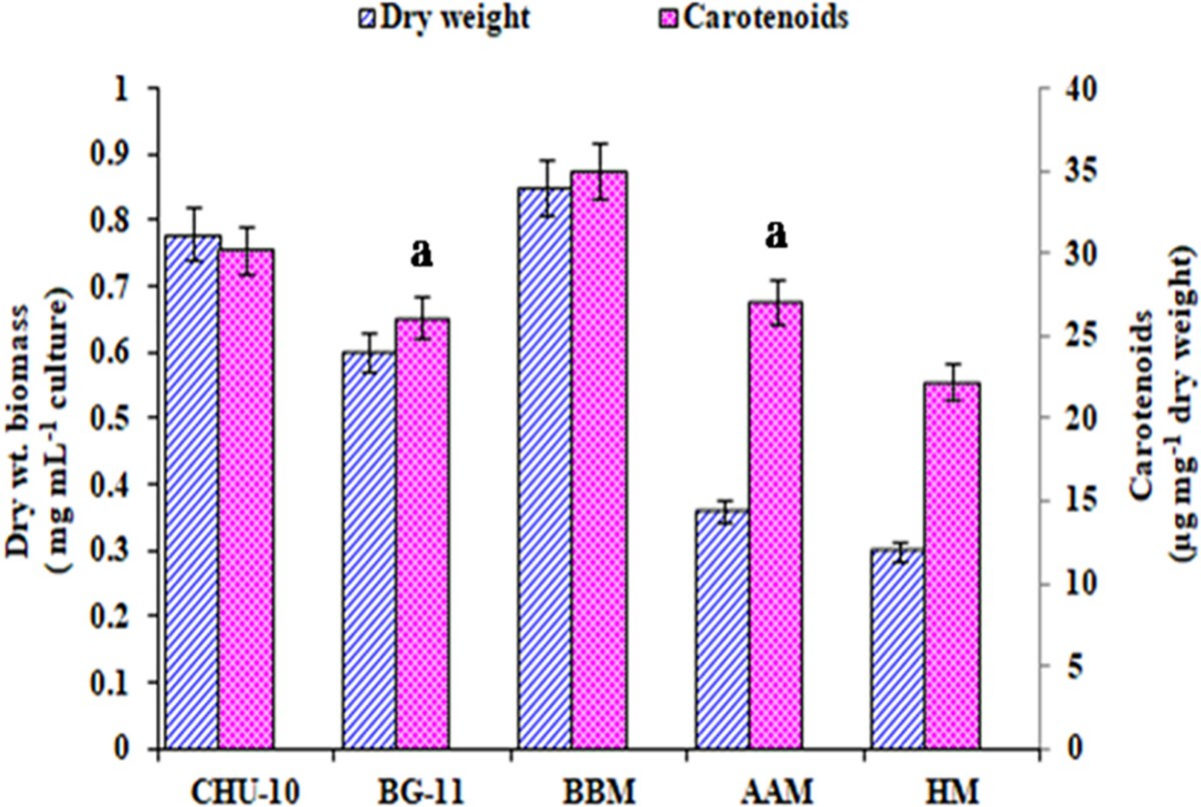
Growth and carotenoid production by *Asterarcys quadricellulare* in different growth media on day 24. Data of both parameter in each medium are significantly different from each other at 95% confidence level (p ≤ 0.05) except for data with same alphabet. n: 6, error bar: SD.

**Fig 5:**
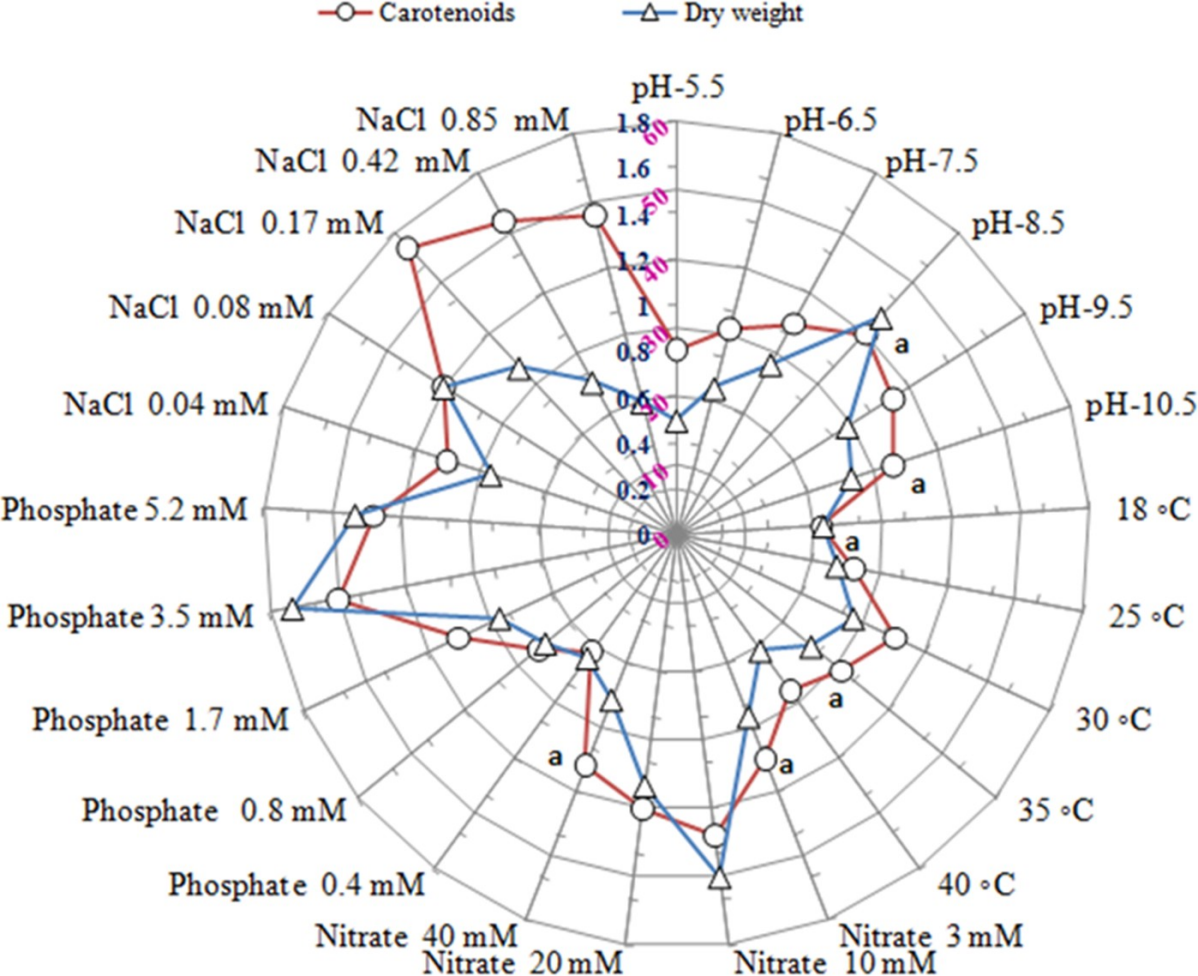
Growth (dry weight biomass mg mL^-1^ culture) and carotenoid (μg mg^-1^ dry weight biomass) production by *Asterarcys quadricellulare* PUMCC 5.1.1 in BB medium under varied culture conditions on day 24. Control conditions: BB medium contained 3 mM NaNO_3,_ 1.7 mM phosphate, 0.04 mM NaCl, pH 7.5 and incubated in white light with 40 μE light intensity (14:10 h light/dark). Data in each parameter are not significantly different from each other at 95% confidence level (p ≤ 0.05). n: 6, error bar: SD.

The growth of test microalga increased with time up to 24 days and afterwards slightly decreased. Carotenoid content in the cells of the organism on the other hand remained almost same up to 20 day and showed significant increase on 24 day and remained almost same afterwards (Fig 3). It indicated that maximum biomass and carotenoid were achieved in the cultures during stationary phase. Thus for optimization of growth and carotenoid production by the test organism day 24 was selected. The growth and carotenoid production by *A*. *quadricellulare* in five different nutrient growth media is shown in Fig 4. On day 24, the test microalga exhibited maximum growth (0.85 mg dry weight mL^-1^) and carotenoid content (35 μg mg^-1^ dry weight biomass) in BB medium. The growth and carotenoid content of the test organism was further assessed by varying pH of BB medium, incubation temperature, quantity of nitrogen source, phosphate and NaCl. All the above modifications of the medium altered the biomass production and cellular carotenoids content (Fig 5). The results revealed that the maximum biomass density (1.6 mg dry weight biomass mL^-1^) was achieved when the organism was grown in basal medium containing 3.5 mM phosphate. This was followed by medium with 10 mM nitrate, followed by basal medium with pH 8.5, followed by 0.08 mM NaCl, while change in temperature other than control cultures did not support more growth. It was interesting to note that all the culture conditions, except for NaCl concentration, which supported more growth of the organism, also supported more carotenoid production. While 0.08 mM NaCl supported more growth, more amounts of carotenoids were produced by the organism when grown in 0.17 mM NaCl containing medium having alkaline pH 8.5 at 30°C. Of the varied concentrations of nitrate and phosphate, the BB medium with 10 mM nitrate (1.5 mg dry biomass mL^-1^) or 3.5 mM phosphate (1.6 mg dry biomass mL^-1^) when added separately resulted in maximum growth of the test microalga. The addition of 0.08 mM NaCl in medium as salinity supported maximum growth of test organism equivalent to 1.2 mg dry biomass mL^-1^ culture (Fig 5).

**Fig 6:**
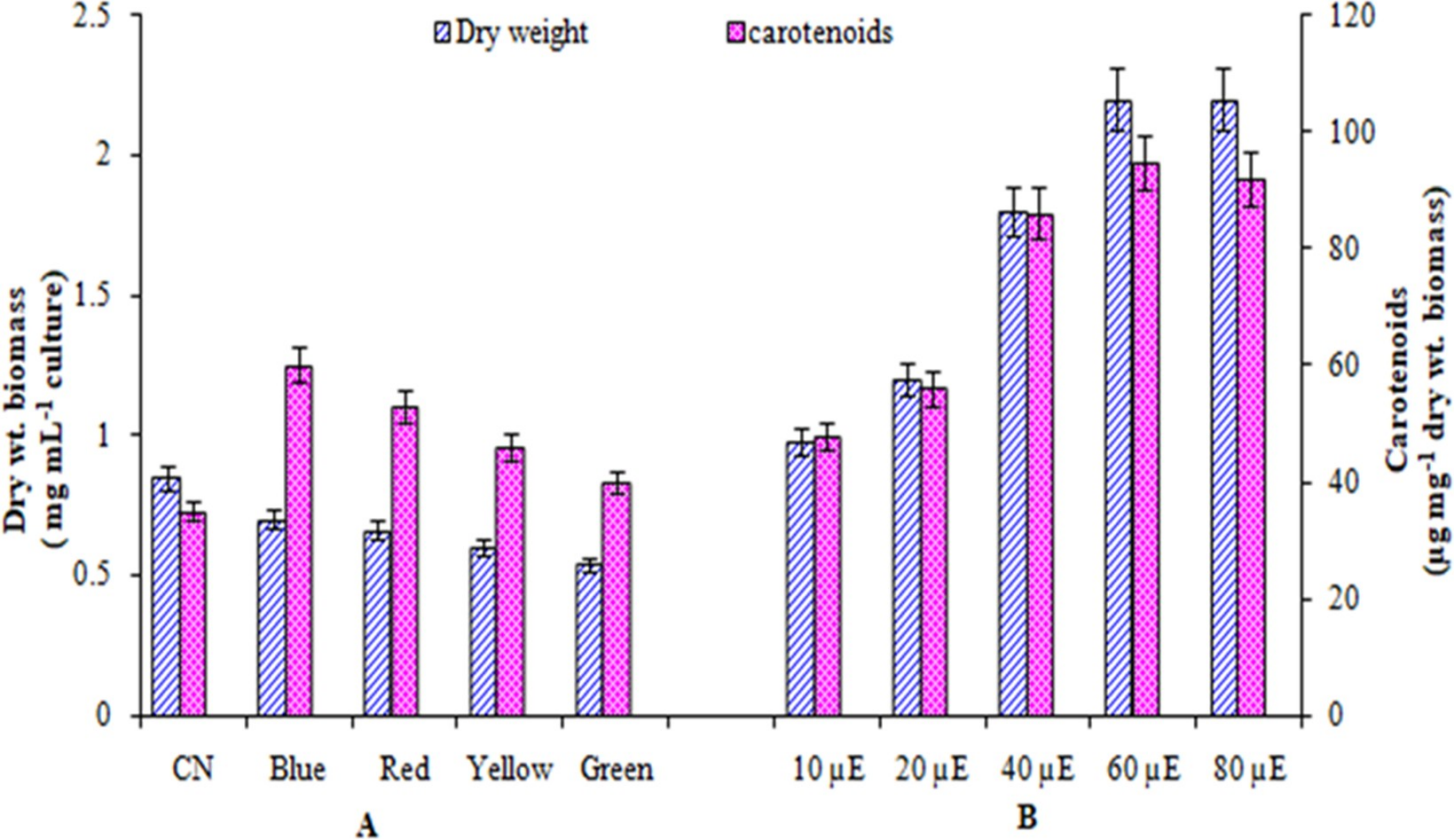
Growth and carotenoid production by *Asterarcys quadricellulare* PUMCC 5.1.1 in BB medium under different light conditions (A) and under different light intensties (B) on day 24. Control conditions: BB medium contained 3 mM NaNO_3,_ 1.7 mM phosphate, 0.04 mM NaCl, pH 7.5 and incubated in white light with 40 μE light intensity (14:10 h light/dark). Conditions for Fig 6B were same as for control except that cultures were incubated in continuous light. Data of both parameter are significantly different from each other at 95% confidence level (p ≤ 0.05). n: 6, error bar: SD.

**Fig 7:**
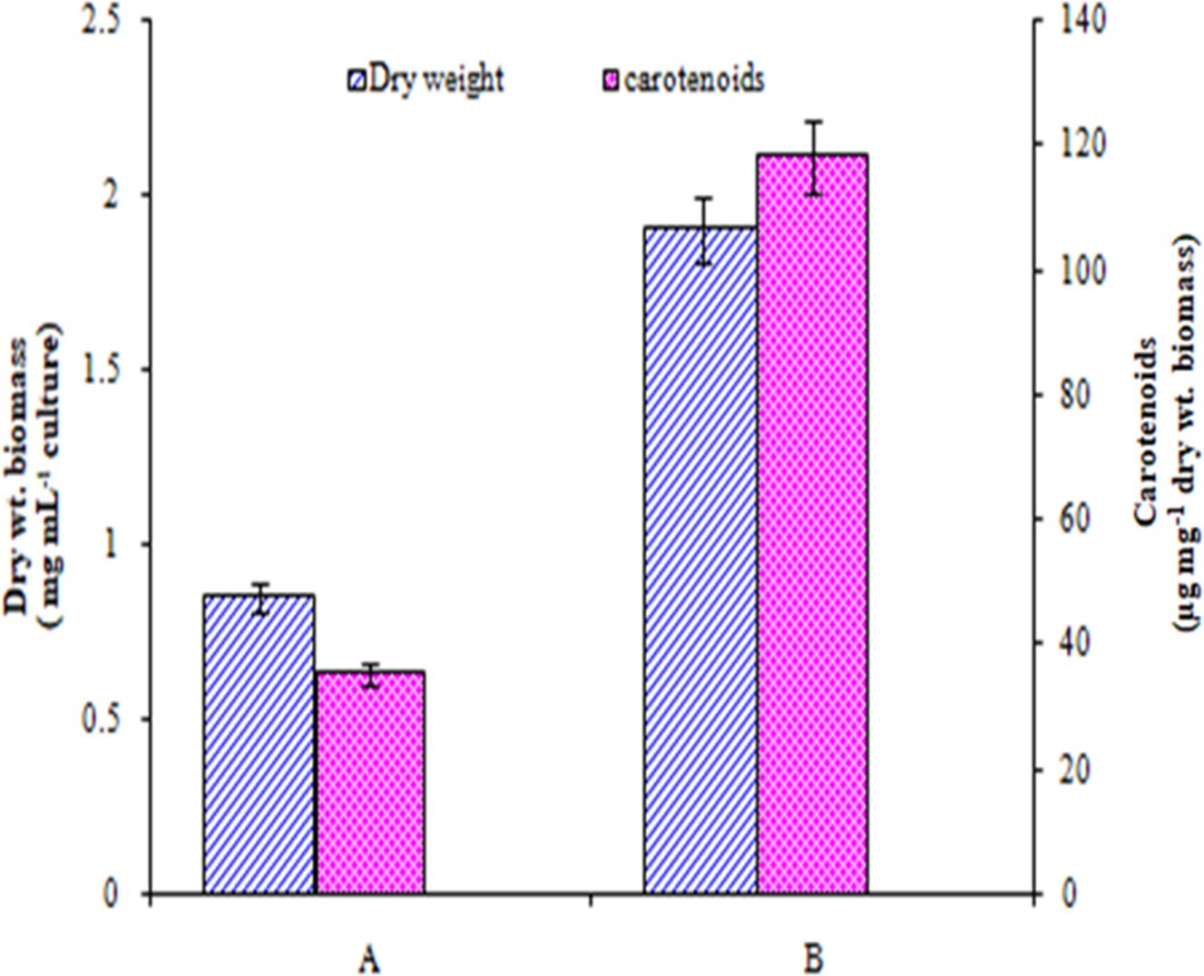
Synergistic effect of optimized conditions on growth and carotenoid production by *Asterarcys quadricellulare* PUMCC 5.1.1 on day 24. A: Control conditions: BB medium contained 3 mM NaNO_3,_ 1.7 mM phosphate, 0.04 mM NaCl, pH 7.5 and incubated in white light with 40 μE light intensity (14:10 h light/dark). B: BB medium contained 10 mM NaNO_3,_ 3.5 mM phosphate, 0.17 mM NaCl and pH 8.5, and incubated in blue light. Data with each condition are significantly different from each other at 95% confidence level (p ≤ 0.05).

The incubation of cultures in red, blue, green or yellow light (14:10 h light/dark) although resulted in less growth than control cultures (under white light) but these lights supported more carotenoid production than control cultures, maximum increase (71%) being in cultures incubated in blue light (Fig 6A). Further, when the cultures were incubated in white light with different intensity, the growth and carotenoids production increased with increase in intensity of light up to 60 μE afterwards these parameters remained same (Fig 6B). The growth and carotenoids under the above condition were 158% and 168% more than control cultures, respectively (Fig 6A & Fig 6B). Synergistic effect of optimized culture conditions also exhibited a significant positive effect on growth and carotenoid production of this microalga (Fig 7).

### Identification and purification of carotenoids

**Fig 8:**
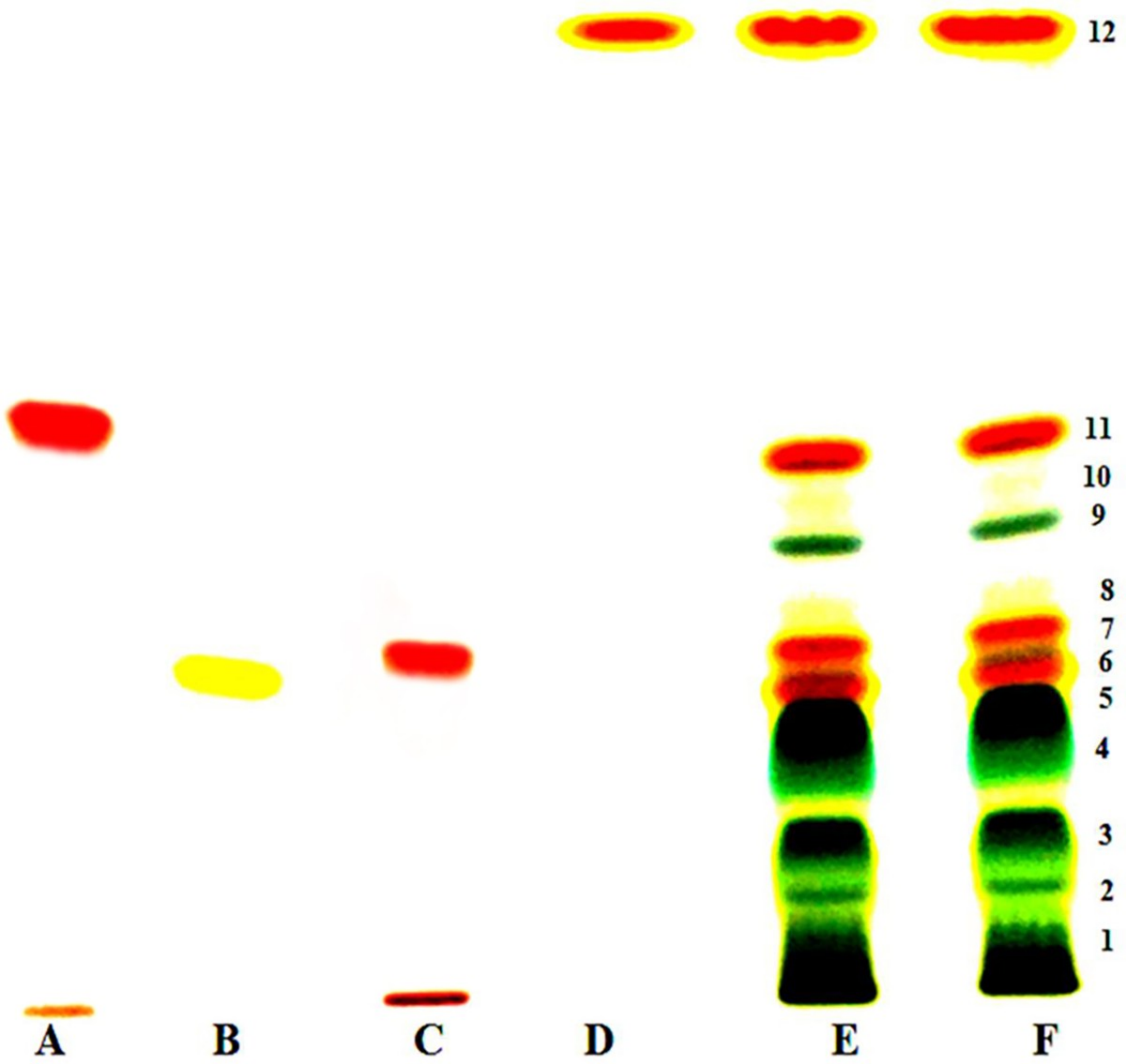
HPTLC profiling of carotenoids in *Asterarcys quadricellulare* grown under synergistic conditions (culture conditions were same as in Fig 7B). Standards: A; Canthaxanthin, B; Lutein, C; Astaxanthin, D; β-carotene, E and F; Cell extract.

**Fig 9:**
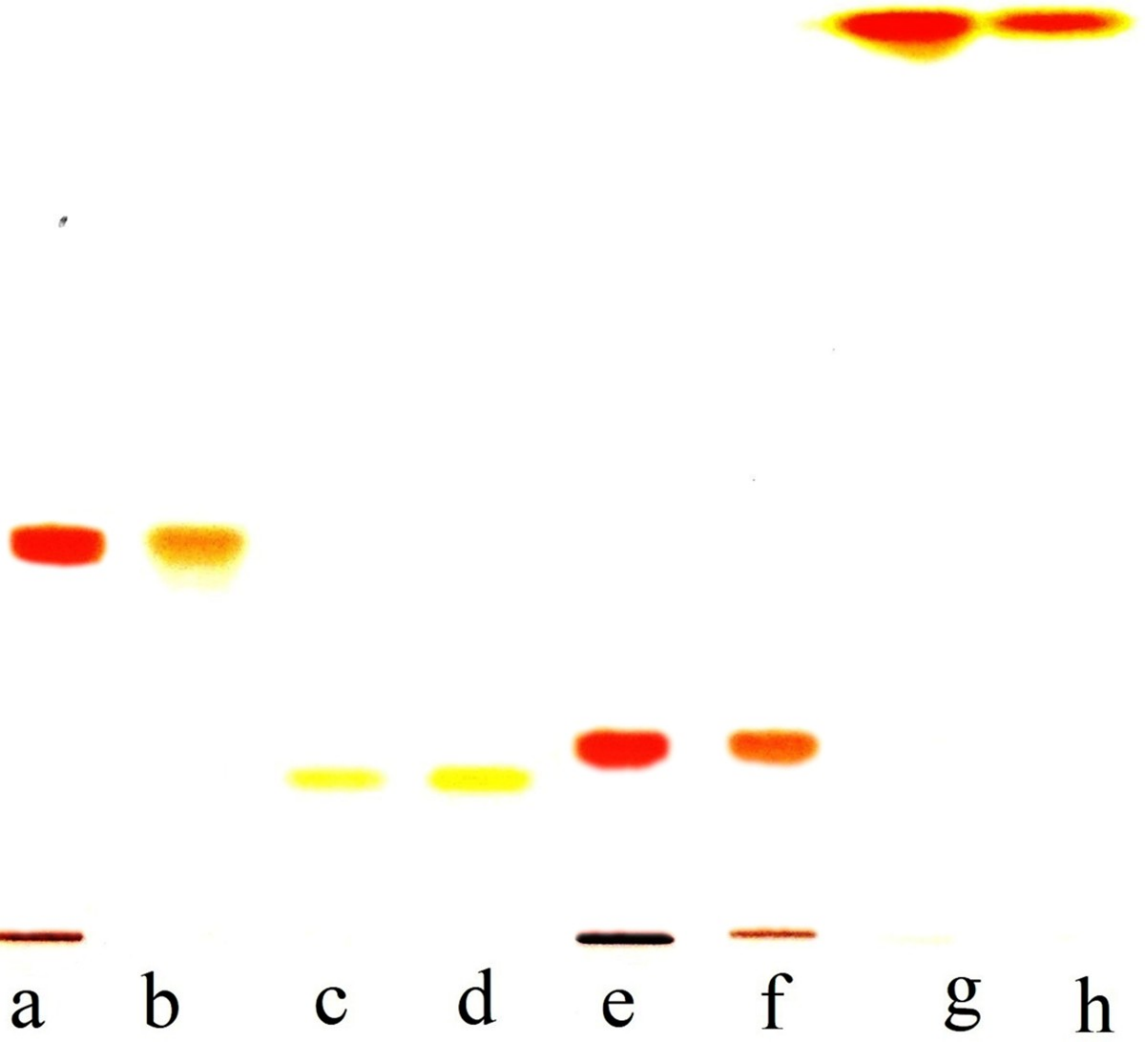
HPTLC profiling of carotenoids in *Asterarcys quadricellulare* purified by flash chromatography. A: standard Canthaxanthin; B: Fraction No. 10; C: standard Lutein, D: Fraction No. 4; E: standard Astaxanthin; F: Fraction No. 12; G. standard β-carotene; H: Fraction No. 5.

**Fig 10:**
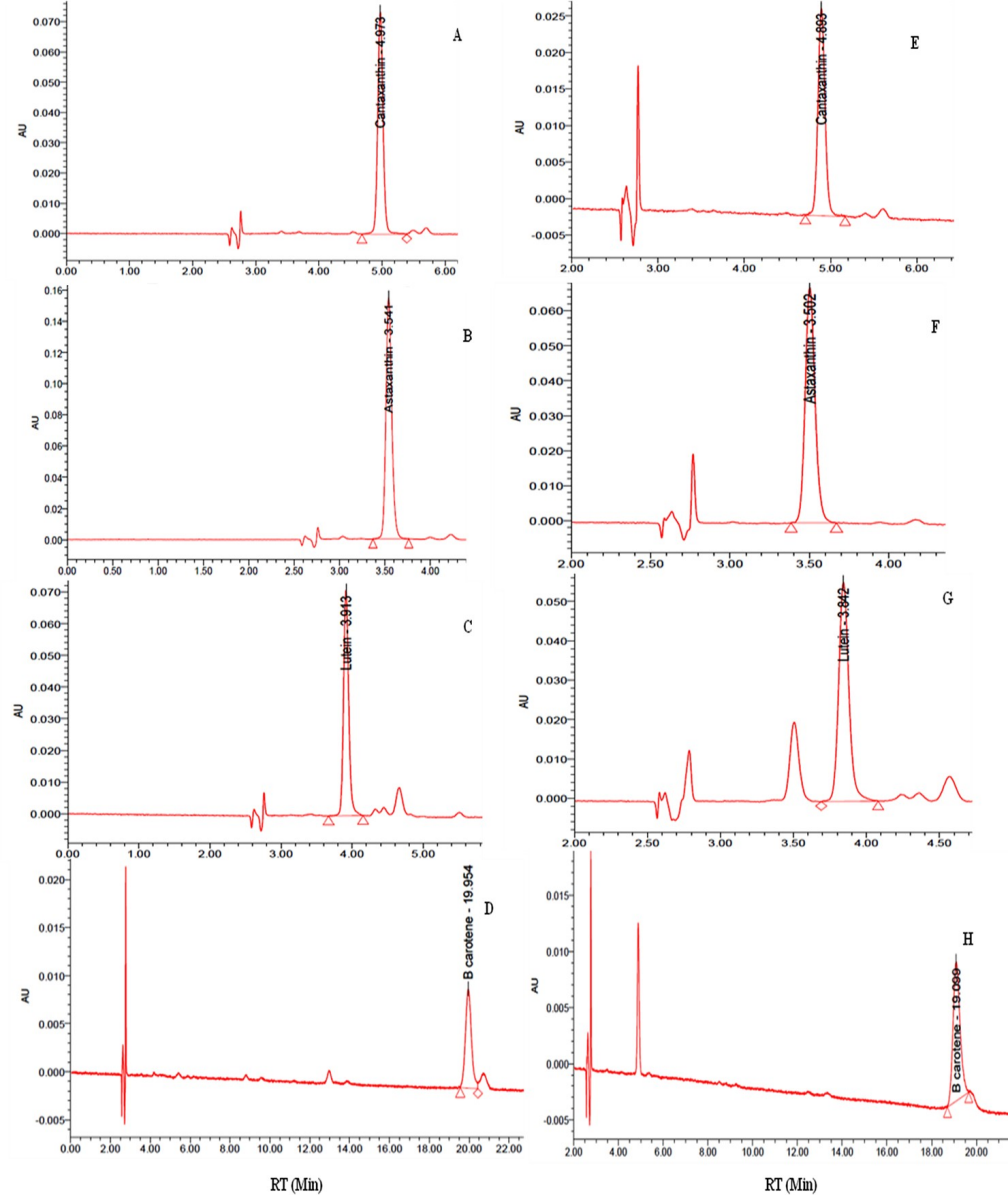
HPLC chromatograph of carotenoids of *Asterarcys quadricellulare* purified by flash chromatography. A: standard Canthaxanthin; B: standard Astaxanthin; C: standard Lutein; D: standard β-carotene; E: Fraction No. 10; F: Fraction12; G: Fraction No. 4; H: Fraction No. 5.

HPTLC chromatogram of crude extract of carotenoids revealed total 12 bands. Of these, four bands: number 7, 8, 11 and 12 matched with the bands of standard astaxanthin, lutein, canthaxanthin and β-carotene, respectively (Fig 8). Further, the intensity of these bands was quite high compared to other bands indicating their good amount. The crude extract was also subjected to flash chromatography for separation and purification of carotenoids. A total of 13 fractions, each of 8 mL, were obtained and subsequently concentrated to dryness under reduced pressure and each was dissolved in 1 ml methanol. Each fraction was subjected to HPTLC individually along with known available standards for identification of carotenoids. Of these, carotenoid bands of fraction 4, 5, 10 and 12 matched with bands of standard lutein, β-carotene, canthaxanthin and astaxanthin, respectively (Fig 9). Thus separation of carotenoids through flash chromatography also confirmed presence of lutein, β-carotene, canthaxanthin and astaxanthin in the crude extract. The identification of these purified carotenoids was further confirmed by HPLC. The peaks of purified canthaxanthin, astaxanthin, lutein and β-carotene appeared at 4.89, 3.50, 3.84 and 19.09 min, respectively, after injection of the sample and exactly matched with known standards (Fig 10). Analysis of peak areas of the HPLC chromatograms revealed that test organism produced 47.0, 15.5, 28.7 and 14.0 μg β-carotene, lutein, astaxanthin and canthaxanthin mg^−1^ dry weight biomass, respectively, on day 24 under combined optimized culture conditions.

## Discussion

Despite enormous diversity in microalgae, only *Dunaliella salina* and *Haematococcus pluvialis* are commercially exploited for carotenoids but these microalgae require specific conditions for their growth and for carotenoid production necessitating to screen more microalgae for high production of carotenoids [21]. In this study, we have screened 40 microalgal strains isolated from different habitats for carotenoid production. Among these, isolate FKN 40 exhibited high growth rate and produced maximum amount of carotenoids (S1 Table) and was thus selected for the present study. On the basis of morphological features it was identified as *Asterarcys quadricellulare* and BLASTn results of partial 18S rRNA gene sequence revealed its 100% similarity with *Asterarcys quadricellulare* Comas 75/76 (AF388375) (Fig 2). Hence the present isolate was named as *Asterarcys quadricellulare* PUMCC 5.1.1. The growth and carotenoid production by the test organism was compared at different phases of growth in BG-11 medium. The microalga *A*. *quadricellulare* exhibited maximum growth (0.62±0.03 mg dry weight biomass mL^-1^culture) and carotenoid production (26±1.75 μg carotenoid mg^-1^ dry weight biomass) on day 24 (Fig 3). Several strategies have been adopted to improve the microalgal growth and development [36, 37]. Mostly the nutrient composition, temperature and light affect the growth and biochemical composition in microalgae [38–42]. Since nutrient requirements of the test microalga for growth are not reported earlier, thus the growth of the test organism was compared in different nutrient media and BB medium supported maximum growth (0.85 mg dry weight mL^-1^ culture) and carotenoids (35 μg mg^-1^ dry weight biomass) of the organism (Fig 4). Paliwal et al. [38] reported maximum growth of cyanobacterium *Synechocystis* sp. CCNM 2510 in Zarrouk’s medium.

It is well known that optimum physical as well as nutrient parameters such as pH of the nutrient medium, temperature, nitrogen and phosphorus source, salinity and light conditions improved growth of microalgae [21–23]. During the present study, these conditions were optimized by varying one parameter at a time keeping others constant. Optimum culture conditions for growth and carotenoid production by the test organism were: pH 8.5, temperature 30 ^o^C, nitrate 10 mM, phosphate 3.5 mM, and NaCl 0.08 mM for growth while 0.17 mM NaCl for carotenoid production (Fig 5). Most of the fresh water microalgae generally prefer slightly alkaline pH [23, 43–45]. *Dunaliella salina*, *Scenedesmus almeriensis* and *Spirulina* sp. also produced high amounts of carotenoids in alkaline pH [45–47]. Temperature is another important factor which affects the growth of microalgae. Most of the mesophilic microalgae have optimum temperature for growth in the range of 25–35 ^o^C [48–49]. In our study, the test organism exhibited maximum growth at 30–35 ^o^C while at temperature below or above this range, growth of the test organism decreased (Fig 5). It is difficult to maintain temperature in outdoor open cultivation but temperature can be effectively managed in closed systems [50]. It has also been suggested that during dark period temperature of the cultures should be lowered to avoid biomass loss due to dark respiration [51] but better biomass has been obtained when same temperature is maintained throughout the cultivation period [50–52].

Nitrogen and phosphorus along with carbon are other important nutrients required for algal growth [53]. The BB medium containing 10 mM nitrate or 3.5 mM phosphate almost doubled the growth of test microalga over the growth of the organism in control cultures (BB medium with 3 mM nitrate and 1.7 mM phosphate (Fig 5). It indicated that contents of nitrate and phosphate in the BB basal medium are not optimal for growth of the organism. These concentrations of nitrate and phosphate when added individually in the medium resulted in 1.25 and 1.5 fold increase, respectively, in the carotenoid production by the organism (Fig 5). The supplementation of nitrate (20–50 mM) and phosphate (3.5 mM) in basal medium significantly improved the growth of *Chlorella vulgaris*, *Dunaliella tertiolecta* and *Haematococcus pluvialis* [54–56]. In autotrophic organisms, nitrogen is important factor influencing intracellular accumulation of carotenoids [2, 57, 58]. As compared to nitrogen, phosphate concentration in medium plays much less role in carotenoid production in microalgae [59]. But our results suggest that nitrate as well as phosphate have almost equal role in increasing carotenoid production in the presently studied organism. Salinity is another important environmental factor affecting carotenoid accumulation in algae [59].When NaCl (0.17 mM) was added as a source of salinity to the cultures of test organism, it improved growth and carotenoid production significantly (Fig 5). However, in contrast to our observations, salinity did not increase the growth of *Scenedesmus* sp. LX1 and *Botryococcus braunii* [60, 61]. The supplementation of NaCl in cultures of *Muriellopsis* sp., *Chlorella zofingiensis* and *Scenedesmus almeriensis* significantly enhanced the level of carotenoids [2, 16, 62].

Light quality, duration and intensity are important factors influencing photosynthesis and biomass yield of microalgae [33, 63, 64]. Incubation of cultures of test organism continuously in 60 μE light increased its growth by 2.58 folds (Fig 6). Daliry et al. [43] reported maximum growth rate of *Chlorella vulgaris* at light intensities of 5000–7000 lux. Wu et al. [44] have also reported high biomass production in *Dunaliella salina* cultures in white light with light intensity of 98 μmol m^-2^ s^-1^ photon flux. Different colours of light did not support the growth of test organism up to the level of growth achieved in control cultures kept in white light (Fig 6A) as reported for other microalgae [33,65]. This is expected as white light has a range of radiations required for optimal photosynthesis. Carotenoid production in the presently studied organism was maximum in blue light (1.7 fold increase compared white light) while the growth was maximum when cultures were grown in white light (Fig 6A). Further, the production of carotenoids increased by 2.4 fold in continuous light compared with discontinuous light. Similarly carotenoid production increased with increase in light intensity up to 60 μE (Fig 6B). It has been suggested that light plays a key role in carotenoid biosynthesis through light signal sensing and downstream regulation [66–67]. The increased intensity and quality of light can bring significant changes in the chemical composition of microalgae leading to increased carotenoid production [68–70]. The increase in production of carotenoids in blue light in this microalga might be due to the fact that it absorbs blue region of spectrum more efficiently [71]. Synergistically, the optimized conditions exhibited positive effect on the growth (from 0.85 to 1.9 mg dry weight biomass mL^-1^ culture) as well as carotenoid production (from 35 to 118 μg mg^-1^ dry weight biomass) of the test organism (Fig 7). The above discussion indicated that each microalgal species has its own nutritional and physical requirements, and to get maximum biomass of any organism these requirements must be determined. Thus, while designing the strategies to achieve higher biomass, combination of pH of medium, temperature, light and optimum concentration of nitrates and phosphate must be taken into consideration.

Several kinds of carotenoids are produced by microalgae. Most of these have been identified and quantified but sometimes only the percentage of the total carotenoid yield is reported [71–74]. HPTLC chromatogram of crude cell extract and fractions of flash chromatography during the present study showed a total of 12 carotenoid bands. Of these, band numbers 7, 8, 11 and 12 matched with the bands of standard astaxanthin, lutein, canthaxanthin and β-carotene, respectively (Figs 8 and 9), but the remaining bands could not be identified due to non availability of all standards of carotenoids. The HPLC chromatogram of flash chromatography purified carotenoids further reconfirmed the presence these four carotenoids (Fig 10). The analysis of HPLC chromatograms revealed that the test microalga produced 47.0, 15.5, 28.7 and 14.0 μg β-carotene, lutein, astaxanthin and canthaxanthin, respectively, mg^−1^ dry weight biomass. These four carotenoids totalling to 105 μg mg^-1^ dry weight biomass represent 89% of the total carotenoids (118 μg mg^-1^ dry weight). Schubert et al. [74] have identified two carotenoids zeaxanthin (97.4%) and β-carotene (2.6%) in microalga *Porphyridium cruentum*. The microalga *Coelastrella strialata* accumulated canthaxanthin, astaxanthin and β-carotene in the cells to the extent of 47.5, 1.5 and 7 mg g^-1^ dry weight [17]. Gong and Bassi reported the production of 11.98 mg lutein g^−1^ dry weight biomass day^−1^ in *Chlorella vulgaris* UTEX 265 in a photobioreactor [75]. Emeish [76] discussed the market value of carotenoids about 3000 US$/kg isolated from green alga *Dunaliella salina*. In conclusion, the test microalga *A*. *quadricellulare* PUMCC 5.1.1 produced 35 μg carotenoids mg^-1^ dry weight biomass under control conditions which get increased to 118 μg carotenoids mg^-1^ dry biomass under optimized conditions. The amounts and rate of carotenoid production by microalgae reported in literature is difficult to compare as different workers have reported these values as percent/per litre/per cell or per day per unit area. The amount of total carotenoid produced by the selected microalga is comparable to *Dunaliella salina* and is significantly higher than the amount of carotenoids produced by other strains of microalgae (Table 1). Of the identified carotenoids, β-carotene accounted for 47% of total carotenoids in the presently studied organism. Thus, the test microalga can be considered as a good candidate for the production of carotenoids especially β-carotene.

**Table 1:** Comparison of carotenoids produced by microalgae reported by various workers.

Sr No.	Organism	Total Carotenoids (μg mg^-1^ dw)	Optimized conditions/ culture age (ca)	Reference
1	*Asterarcys quadricellulare*	118	BB medium, pH: 8.5, Temp: 30°C, Nitrate: 10 mM, Phosphate:, 3.5 mM, NaCl: 0.17 mM Light: 80 μmol photon m^-2^ s^-1^ ca: 24 d	Present study
2	*Coelastrella striolata*	56.0	BB-medium, Nitrate: 500 mg L^-1^, Temp: 25°C, Light: 65 μmol photon m^-2^ s^-1^ ca: 30 d	[17]
3	*Dunaliella salina*	100 (102.5 mg m^-2^ d^-1^)	F2 medium, Temp: 25°C, pH: 7.5, Light: 281 μmol photon m^-2^ s^-1^ ca: 10 d	[45]
4	*Dunaliella* sp.	26 pg cell ^-1^	Johnson medium, Temp: 20 ^o^C, pH: 7.0, NaCl: 20% ca: 39 d	[77]
5	*Haematococcus pluvialis*	1.6 mg L^-1^	Bristol medium, 23 ^o^C, Light: 85 μmol photon m^-2^ s^-1^ ca: 13 d	[78]
		98	BAR medium, 28 ^o^C, Temp: 28°C, Light: 345 μmol photon m^-2^ s^-1^ Ca: 14 d	[79]
		1.6 mg L^-1^ d^-1^	Standard inorganic medium (strength: x0.3), Temp: 20°C, Light: 2500 μmol photon m^−2^ s^−1^ ca: 12 d	[52]
6	*Vischeria helvetica*.KGU-Y001	11.50	BB medium, 25 ^o^C, Light: 214 μmol photon m^-2^ s^-1^ ca: 20 d	[80]

## Supporting information

S1 Table Growth and amount of carotenoids produced by selected microalgae on day 8. (DOCX)
