# Supplementary material for: High production of carotenoids by the green microalga Asterarcys quadricellulare PUMCC 5.1.1 under optimized culture conditions
Source: PLoS One. 2019 Sep 6;14(9):e0221930. doi: 10.1371/journal.pone.0221930 (PMC6730905; doi:10.1371/journal.pone.0221930)
Supplement: S1 Table — (DOCX) [file pone.0221930.s001.docx]

**S1 Table. Growth and amount of carotenoids produced by selected microalgae on day 8**

| **S No** | **Organism** | **Strains** | **Growth rate (h^-1^)** | **Carotenoid content**  **(µg mg^-1^ dry wt.)** | **S No** | **Organism** | **Strains** | **Growth rate (h^-1^)** | **Carotenoid content**  **(µg mg^-1^ dry wt.)** |
| --- | --- | --- | --- | --- | --- | --- | --- | --- | --- |
| **1** | *Chlorella vulgaris* | BNL54 | 0.0065 | 11.0 ± 0.37 | **21** | *Asterarcys quadricellulare* | FKN44 | 0.0071 | 22.0 ± 1.37 |
| **2** | *Chlorella vulgaris* | RFH2 | 0.0077 | 25.0 ± 1.19 | **22** | *Chlorella vulgaris* | CLS48 | 0.0071 | 21.0 ± 1.27 |
| **3** | *Chlorella* sp.1 | FRL34 | 0.0062 | 17.9 ± 1.14 | **23** | *Scenedesmus quadricauda* | RFJ25 | 0.0068 | 18.6 ± 1.34 |
| **4** | *Scenedesmus quadricauda* | CLS110 | 0.0062 | 12.2 ± 0.67 | **24** | *Scenedesmus quadricauda* | RFJ16 | 0.0068 | 22.3 ± 1.23 |
| **5** | *Scenedesmus quadricauda* | CLS111 | 0.0071 | 19.0 ± 1.36 | **25** | *Asterarcys quadricellulare* | FKN4 | 0.0059 | 21.6 ± 1.37 |
| **6** | *Scenedesmus quadricauda* | FRL70 | 0.0071 | 21.0 ± 1.86 | **26** | *Asterarcys quardicellulare* | FKN46 | 0.0074 | 21.0 ± 1.38 |
| **7** | *Scenedesmus quadricauda* | FRL68 | 0.0068 | 22.2 ± 1.86 | **27** | *Chlorella*  sp. | FRL37 | 0.0074 | 12.0 ± 0.28 |
| **8** | *Scenedesmus quadricauda* | PWJ97 | 0.0068 | 21.6 ± 1.18 | **28** | *Desmodesmus armatus* | RFJ24 | 0.0074 | 21.0 ± 1.18 |
| **9** | *Scenedesmus dimorphus* | RFJJ96 | 0.0065 | 19.0 ± 1.11 | **29** | *Chlorococcum* sp*.* | SWH33 | 0.0059 | 18.6 ± 1.32 |
| **10** | *Scenedesmus dimorphus* | RFJ27 | 0.0059 | 22.2 ± 1.18 | **30** | *Chlorella* sp. | RFH9 | 0.0062 | 10.0 ± 0.39 |
| **11** | *Chlorococcum infusiform* | CLS41 | 0.0059 | 15.0 ± 1.17 | **31** | *Desmodesmus armatus* | FRL58 | 0.0065 | 22.2 ± 1.38 |
| **12** | *Chlorococcum infusiform* | FKN56 | 0.0053 | 16.0 ± 1.76 | **32** | *Coelastrum elegan* | FKN53 | 0.0062 | 18.7 ± 1.38 |
| **13** | *Chlorococcum infusiform* | PWH47 | 0.0053 | 17.5 ±1.18 | **33** | *Chlorella vulgaris* | RFH 8 | 0.0068 | 21.0 ± 2.37 |
| **14** | *Coelastrum. micoporum* | CLS39 | 0.0059 | 19.6 ± 1.27 | **34** | *Desmodesmus armatus* | CLS108 | 0.0071 | 21.6 ± 1.39 |
| **15** | *Coelastrum micoporum* | CLS109 | 0.0059 | 20.0 ± 1.36 | **35** | *Chlorella vulgaris* | FKN59 | 0.0074 | 19.4 ± 1.16 |
| **16** | *Coelastrum elegan* | FKN52 | 0.0062 | 18.8 ± 1.18 | **36** | *Scenedesmus quadricauda* | RFJ26 | 0.0068 | 21.8 ± 2.28 |
| **17** | *Chlorella vulgaris* | RFJ 18 | 0.0071 | 21.0 ± 1.26 | **37** | ***Asterarcys quadricellulare*** | **FKN 40** | **0.0083** | **28.3 ± 1.4** |
| **18** | *Desmodesmus armatus* | CLS 50 | 0.0074 | 21.0 ± 1.38 | **38** | *Chlorella vulgaris* | PWJ98 | 0.0071 | 21.0 ± 1.39 |
| **19** | *Chlorella vulgaris* | BNL55 | 0.071 | 23.2 ± 1.48 | **39** | *Scenedesmus dimorphous* | RFJ16 | 0.0071 | 21.0 ± 2.38 |
| **20** | *Scenedesmus quadricauda* | RFJ17 | 0.0079 | 26.8 ± 1.28 | **40** | *Scenedesmus quadricauda* | RFJ98 | 0.0071 | 21.3 ± 2.36 |

The strains were grown in BG-11 medium for 8 d and amount of carotenoids was determined. Data are means ± SD of three replicate.
